# Characterization of growth differentiation factor 15 (GDF15) as a neurotropic adipokine permeable to the brain

**DOI:** 10.1038/s41598-025-14846-8

**Published:** 2025-08-17

**Authors:** Andreas Schmid, Emily Wilfurth, Alexandra Höpfinger, Manuel Behrendt, Edita Islami, Thomas Karrasch, Martin Berghoff, Andreas Schäffler

**Affiliations:** 1 Department of Internal Medicine III, Giessen University Hospital, Giessen, Germany; 2 Department of Neurology, Giessen University Hospital, Klinikstraße 33, 35392 Gießen Giessen, Germany

**Keywords:** GDF15, Cerebrospinal fluid, Fat-Brain axis, Adipocyte, Adipose tissue, Adipokine, Endocrinology, Neurology

## Abstract

GDF15 (growth/differentiation factor-15) belongs to the superfamily of transforming growth factor-beta. Little is known about adipocytic regulation of GDF15, its concentrations in serum and cerebrospinal fluid (CSF), its permeability to the brain, and its correlation with neurological diseases. This knowledge is important for a potential and clinical role of GDF15 as a mediator of the fat-brain axis in metabolic and neurological diseases. GDF15 mRNA expression in 3T3-L1 adipocytes was measured by qPCR and GDF15 protein levels in supernatants, serum and CSF were determined by ELISA. In vitro, GDF15 expression is nearly absent in pre-adipocytes and strongly upregulated during adipocyte differentiation. Insulin upregulates GDF15 secretion in adipocytes under normo- and hyperglycemic conditions. In vivo, we quantified GDF15 protein concentrations in paired samples of serum and CSF in a large and well-characterized clinical cohort of n = 390 patients undergoing neurological investigation and spinal puncture. This broad data set could serve as a basis for the development of GDF15 reference values in serum and CSF. GDF15 is highly permeable to the brain according to a specific CSF / serum ratio of 306 × 10^–3^. GDF15 is significantly increased in overweight and type 2 diabetic patients and correlates positively with serum glucose and HbA_1c_. GDF15 in CSF is elevated in patients with increased CSF cell count and impaired blood–brain-barrier function. Among five subsets of neurological diagnoses, GDF15 is exclusively increased in CSF and serum of patients with infectious diseases. GDF15 represents a promising adipokine and mediator of the fat-brain-axis that is co-regulated with metabolic factors and elevated in neurological patients with infectious diseases.

## Introduction

GDF15 (growth/differentiation factor-15) belongs to the superfamily of transforming growth factor-beta (TGFβ1) and to the subfamily of the bone morphogenic proteins (BMP)^1^. This pleiotropic secretory protein is induced by inflammatory processes and DNA damage, regulates macrophage function, and is involved in metabolic and atherosclerotic processes^2^ in many cell types such as cardiomyocytes, immune cells, and endothelial cells. Importantly, the interaction of GDF15 with its receptor GFRAL (glial cell derived neurotrophic factor receptor alpha-like) in the central nervous system is able to cause weight loss in diet-induced obesity and in other animal models of obesity via reduction of food^3,4^ and fat^5^ intake. Importantly, metformin was shown to elevate circulating GDF15 levels^6^ in mice and humans.

GDF15 might have a potential role as a secretory mediator protein of the fat-brain axis (adipokine) and the muscle-brain axis (myokine). It is expressed and upregulated in murine white and brown adipocytes by stress response signaling^5,7^, in human pre-adipocytes by caloric restriction-induced weight loss^8^, and in human adipocytes by inhibitors of mitochondrial ATP synthesis^9^. GDF15 expression in muscle is upregulated by exercise and it shows a temperature-dependent regulation after exercise, highlighting an additional role of GDF15 as a myokine/exerkine^10–12^. In general, several secreted adipokines such as adiponectin^13,14^, leptin^15^, chemerin^16^, adipsin^17^, resistin^14,18^, progranulin^18^, CTRP-3^19^, RBP4 (retinol binding protein-4), clusterin, PEDF (pigment epithelium derived factor)^20^ as well as secreted myokines such as meteorin-like protein^21^ represent circulating physiological regulator proteins that are able to cross the blood–brain-barrier (BBB) and thus appear in cerebrospinal fluid (CSF). As provided by the *Human Protein Atlas Project* (https://www.proteinatlas.org/ENSG00000130513-GDF15/tissue), GDF15 mRNA expression is detectable in many organs and also in total adipose tissue. However, expression in total adipose tissue ranges only on the 10^th^ place compared to other organs regarding its quantity. Based on this observation and due to the fact that total adipose tissue contains many different cell types, the exact expression profile of GDF15 mRNA in pre-adipocytes and fully differentiated (mature) adipocytes remains largely unknown, as well as the quantity of secreted protein.

Motivated by these considerations, it was the aim of the present study to investigate the hypothesis that GDF15 represents both, a secreted adipokine of adipocytes and a circulating protein in human serum that is able to cross the BBB. In this context, we aimed to.

- quantify the GDF15 mRNA expression during hormonally-induced differentiation of 3T3-L1 pre-adipocytes into mature adipocytes (an established cell culture model for studying adipocyte biology)
- determine whether secreted GDF15 protein is detectable in supernatants of 3T3-L1 adipocytes
- quantify the protein concentrations of GDF15 in paired samples of serum and CSF in a large and well-characterized clinical cohort of n = 390 patients undergoing neurological investigation and spinal puncture
- calculate specific CSF/serum ratios for GDF15 in order to prove and to characterize the relationship between peripheral serum concentrations and CSF concentrations
- investigate correlations of peripheral and central GDF15 levels with anthropometric, metabolic, and inflammatory parameters in a cohort of patients suffering from a subset of neurological diseases
- find pathophysiological implications of GDF levels and its CSF/serum ratio with specific neurological disorders such as multiple sclerosis, epilepsy, vascular CNS disease, pseudotumor cerebri, and infectious CNS disease.

## Material and methods

### Study population and data collection

The monocentric, tertiary care center, cross-sectional, clinical study cohort was established by the neurological and endocrinological departments of the University Hospital Giessen (Germany) and included a total number of n = 390 patients undergoing neurological evaluation including spinal puncture^16^. Paired samples of venous blood serum and CSF were collected and immediately frozen at −20 °C. Detailed anthropometric data (gender, weight, height, BMI), clinical data (history, medication, use of contraceptives, smoking habits), concomitant diseases (such as type 2 diabetes, dyslipidemia, hypertension), and a plenty of standard biochemical parameters in serum (lipoproteins, glycosylated hemoglobin A1c (HbA_1c_), glucose, CRP, leukocytes, protein, albumin, alanine transaminase (ALT), aspartate transaminase (AST), bilirubin, creatinine, urea, lactate dehydrogenase (LDH)) and in CSF (cell count, protein, albumin, lactate, immunoglobulins, presence of oligoclonal bands) were assessed as described previously^16^. The grade of BBB dysfunction was classified by the neurochemical laboratory unit using standard methods such as CSF/serum albumin ratio. The grading defined four subgroups (none, mild, moderate, severe) of BBB dysfunction and the CSF cell count was divided into two subgroups (< 5 cells/μL; ≥ 5 cells/μL). The detailed clinical parameters of the entire study cohort can be retrieved from our recent study^16^. Briefly, n = 390 study subjects were categorized by a board-certified neurologist assigned to the following subgroups of neurological diagnoses: multiple sclerosis (n = 68), epilepsy (n = 50), cerebrovascular disease (n = 46), infectious disease (n = 30), and pseudotumor cerebri (idiopathic intracranial hypertension) (n = 26). 170 individuals were finally diagnosed as having no neurological disease (exclusion diagnosis) and were included as a control group. All study participants gave informed consent and the study was approved by the local ethical committee (registration note *AZ 13/16*).

### Enzyme-linked immunosorbent assay (ELISA)

GDF15 concentrations in human blood serum and CSF were quantified applying ELISA techniques (DuoSet Development Kit for human GDF15, Bio-Techne; Minneapolis, MN, USA). The detection range was 7.8—500 pg/mL. Murine GDF15 protein levels in cell culture supernatants were measured by ELISA specifically detecting the murine protein (Mouse DuoSet Development Kit, Bio-Techne; Minneapolis, MN, USA) with a detection range of 7.8—500 pg/mL. All measurements were performed in technical duplicates and were repeated in case of an intra-duplicate CV (coefficient of variation) exceeding 20%. In the figures, the respective mean values of the duplicates are given.

### Cell culture of 3T3-L1 adipocytes

3T3-L1 fibroblasts (American Type Culture Collection (ATCC), purchased from LGC Standards, Teddington, UK) were cultured and differentiated into mature adipocytes as described recently^22^. Briefly, cells were cultured at 37 °C and 5% CO2 in Dulbecco´s Modified Eagle Medium (Biochrom AG, Berlin, Germany) supplemented with 10% newborn calf serum (Sigma-Aldrich, Deisenhofen, Germany) and were differentiated into adipocytes in DMEM/F12/glutamate medium (Lonza, Basel, Switzerland) supplemented with 20 µM 3-isobutyl-methyl-xanthine (Serva, Heidelberg, Germany), 1 µM corticosterone, 100 nM insulin, 200 µM ascorbate, 2 µg/ml transferrin, 5% fetal calf serum (FCS, Sigma-Aldrich, Deisenhofen, Germany), 1 µM biotin, 17 µM pantothenate, 100 nM rosiglitazone (all from Sigma Aldrich, Deisenhofen Germany), and 300 µg/ml Pedersen-fetuin (MP Biomedicals, Illkirch, France). Cell morphology was controlled by light-microscopy throughout the process of differentiation. Mature adipocytes were incubated in serum-free DMEM/F12 medium for 24 h prior to stimulation experiments. For experimental settings, concentrations of glucose (1 and 4.5 g/L) and insulin (10 nM) were adjusted in serum-free medium. 1 g/L (equates to 100 mg/dL or to 5.6 mM, respectively) represents a physiological and fasting glucose concentration in humans without diabetes mellitus. 4.5 g/L (equates to 450 mg/dL or to 25 mM, respectively) is a typical glucose value seen in overt and poorly controlled diabetes mellitus. Regarding the relevance of metabolic diseases in the regulation of GDF15, we aimed to address the potential role of glucose concentrations in vitro as published earlier^23–25^.

In order to detect unintended effects on cell viability, LDH activity was quantified in cell supernatants (Cytotoxicity Detection Kit, Roche, Mannheim, Germany).

### GDF15 mRNA expression by quantitative PCR (qPCR)

Total RNA was isolated from mature 3T3-L1 adipocytes applying the RNeasy® Mini Kit (Qiagen, Hilden, Germany). Reverse transcription of RNA (QuantiTect Reverse Transcription Kit from Qiagen, Hilden, Germany) was performed in order to generate corresponding cDNA for quantitative real-time PCR (qRT-PCR) (iTaq Universal SYBR Green Supermix, CFX Connect RT-PCR system; Bio-Rad, Munich, Germany). Target gene mRNA levels were quantified using the following primer sequences:

Murine GDF15: 5’-CCGAGAGGACTCGAACTCAG-3’/5’-TAAGAACCACCGGGGTGTAG-3’.

Murine GAPDH: 5’-TGTCCGTCGTGGATCTGAC-3’/5’-AGGGAGATGCTCAGTGTTGG-3’.

All applied oligonucleotides were purchased from Metabion (Martinsried, Germany). Gene expression levels were quantified applying ddC_T_ method. GDF15 gene expression levels were normalized to expression of murine GAPDH.

### Statistical analysis

Statistical analysis was performed applying the software package SPSS (Version *29.0*; IBM, Armonk, NY, USA). Unrelated groups were compared by Mann–Whitney U-test (n = 2) or Kruskal–Wallis test (n > 2). Pairwise comparison of related samples was performed by Wilcoxon test (n = 2) or Fisher test (n > 2). Bonferroni correction was applied for multiple comparisons. Data regarding comparisons between groups are graphically presented either as bar diagrams (means ± twofold standard error of the mean) or as box plots, with whiskers giving interquartile ranges. In the presented box plot graphs, dots and stars represent outlying values exceeding the interquartile range (IQR) (between 25 and 75 percentile). Dots (circles): mild outliers 1.5 × to 3 × IQR. Stars (asterisks): extreme outliers more than 3 × IQR. Spearman’s rank correlation coefficient was applied to evaluate associations between variables and these correlations are presented as scattered plots. In general, findings with a *P* value < 0.05 were considered statistically significant.

## Results

### Expression, secretion and regulation of GDF15 during adipocyte differentiation

**Fig. 1:**
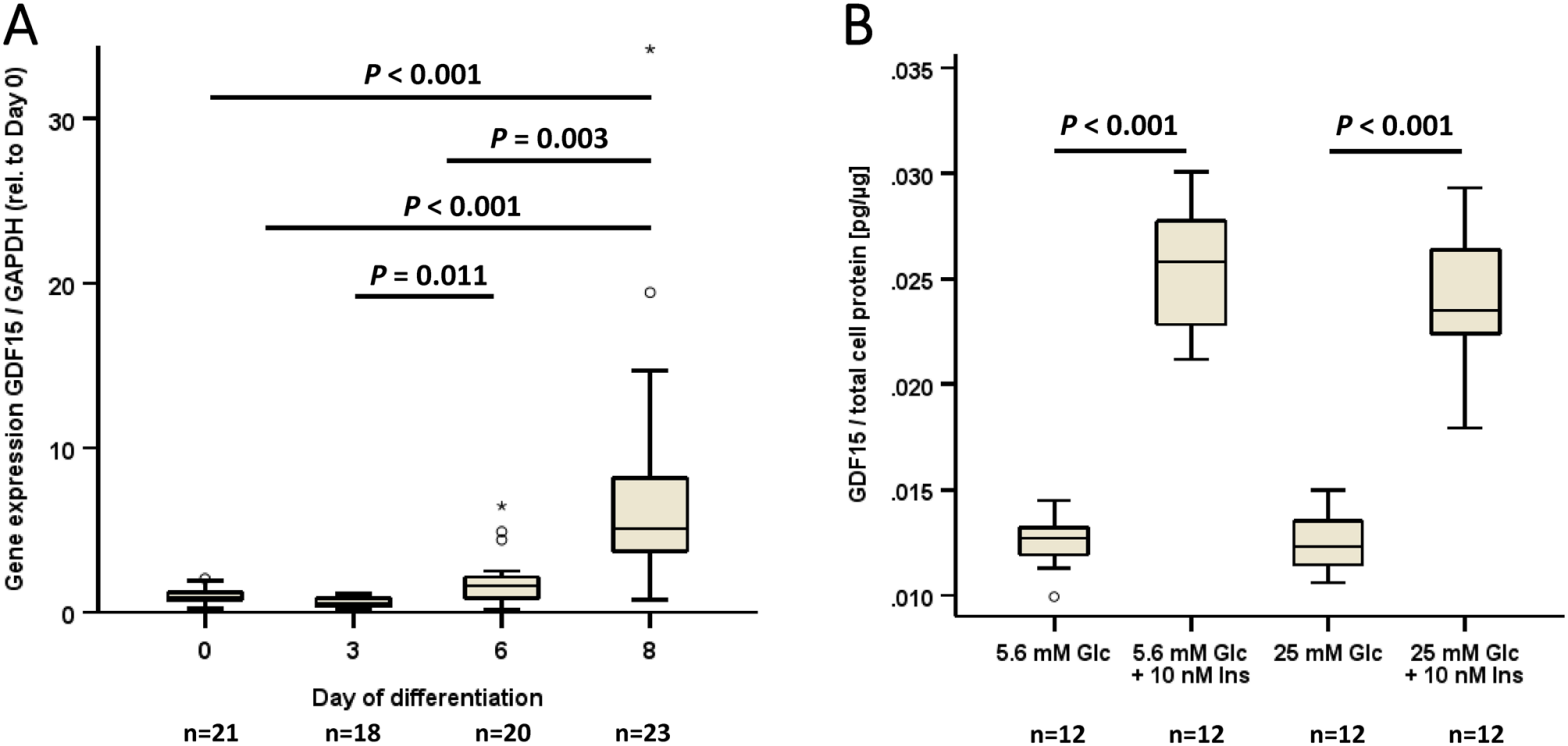
Expression of GDF15 mRNA and secretion of GDF15 protein in 3T3-L1 adipocytes. **Panel A** Upregulation of GDF15 mRNA expression by qPCR during the differentiation of 3T3-L1 pre-adipocytes (day 0) into mature adipocytes (day 8). The Kruskal-Wallis test was performed. **Panel B** Secretion of GDF15 protein into supernatants by ELISA is upregulated by insulin under normo- and hyperglycemic conditions. The Kruskal-Wallis test was performed.

As shown in Fig. 1A, GDF15 mRNA expression by qPCR is very low in pre-adipocytes at day 0 and 3 of hormonally induced differentiation. Expression increases from day 6 of differentiation and is strongly upregulated (about eightfold) in fully differentiated (mature), lipid-loaded adipocytes (*P* < 0.001). GDF15 protein is also detectable by ELISA in supernatants of differentiated adipocytes (Fig. 1B). Since glucose and insulin play an important role in adipocyte differentiation as well as in obese and type 2 diabetic patients, the effect of low glucose vs. high glucose concentrations on GDF15 protein quantity in cell supernatants was investigated by ELISA. High glucose concentrations (25 mM) vs. low (physiological) glucose concentrations (5.6 mM) did not affect GDF15 protein concentrations in the absence of insulin (Fig. 1B). Incubation of cells with 10 nM insulin for 6 h significantly (*P* < 0.001) increased GDF15 secretion into supernatants under low and high glucose conditions (Fig. 1B). Taken together, GDF15 exhibits the typical expression and secretion profile of classical and insulin-sensitive adipokines such as adiponectin and leptin.

### GDF15 quantities in human cerebrospinal fluid and serum

**Fig. 2:**
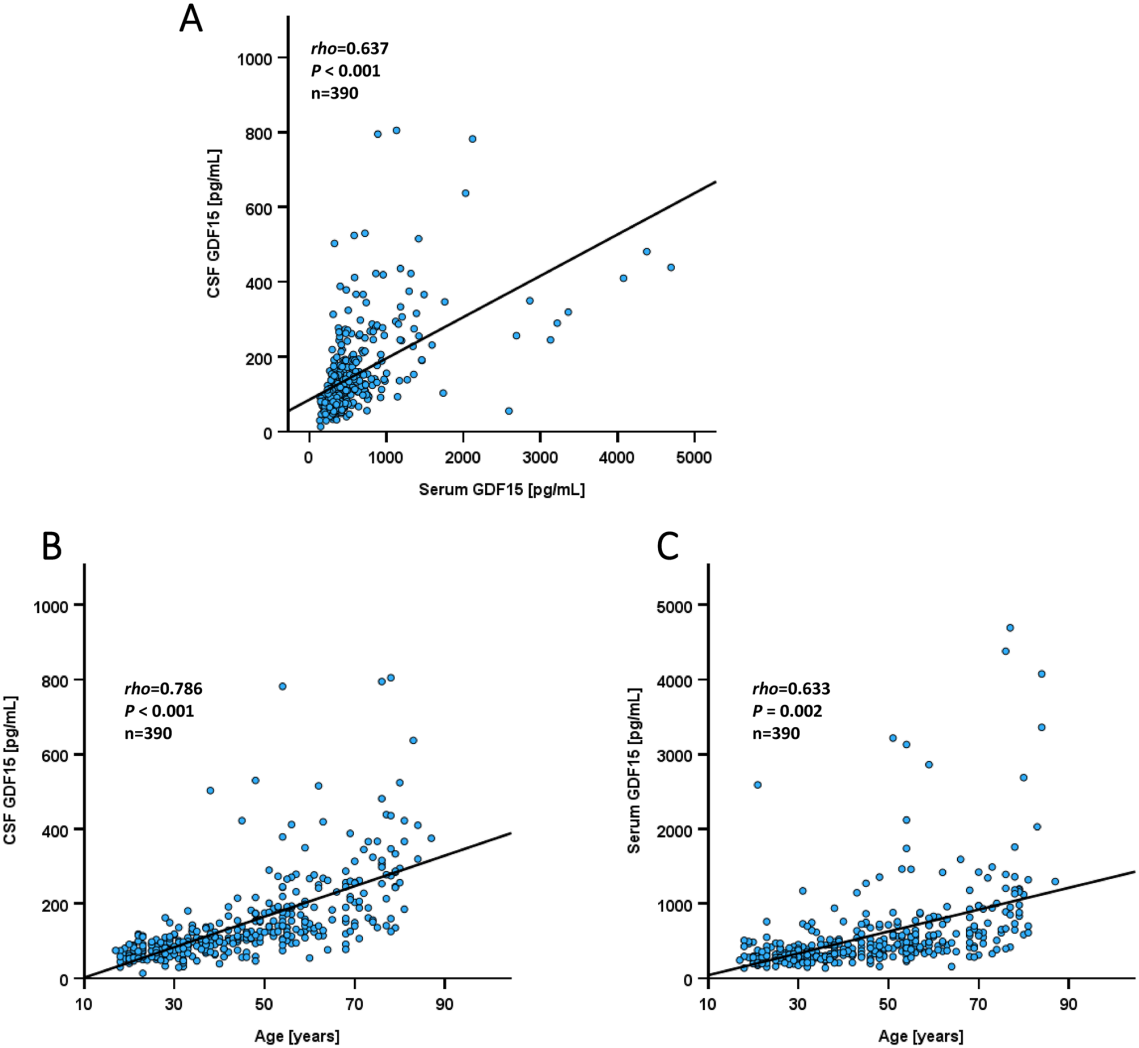
Correlation analysis by scatter plot diagrams. **Panel A** Positive correlation of serum and CSF GDF15 concentrations. **Panel B** Correlation of GDF15 concentrations in CSF with age. **Panel C** Correlation of GDF15 concentrations in serum with age. The Spearman-rho rank correlation test was applied.

GDF15 quantities could successfully be measured in duplicates in serum and CSF of all study participants (Table 1). In the entire study cohort (n = 390), mean serum GDF15 concentrations were 561.01 ± 552.78 pg/mL (range: 135.74–4693.21 pg/mL) compared to CSF mean values of 146.84 ± 111.88 pg/mL (range: 13.10–804.76 pg/mL). Thus, mean serum concentrations are ~ fourfold higher when compared to CSF and this difference is of high statistical significance (p < 0.001). For the first time, a specific CSF/serum ratio of GDF15 could be calculated with a mean value of 0.306 ± 0.008 (range: 0.021–1.536) and should classically be given as 306 × 10^–3^. According to the classical CSF/serum ratio of albumin in *Reiber* diagrams, the GDF15 CSF/serum ratio describes the BBB permeability of GDF15. As presented in Fig. 2A, there is a strong (*P* < 0.001) and positive correlation (*rho* = 0.637) between serum and CSF GDF15 concentrations. Taken together, GDF15 has a relatively high CSF/serum ratio that resembles a high grade of permeability to the brain.

**Table 1:** Characteristics of the study population.

Total cohort: n = 390	Mean ± SD [range]		
Age [y]	45.6 ± 17.9 [17–87]		
Weight [kg]	79.9 ± 20.2 [44.0–190.0]		
Height [cm]	171.3 ± 8.9 [146–194]		
BMI (kg/m^2^)	27.19 ± 6.31 [16.4–62.0]		
Subgroups	n (%)	GDF15 (pg/ml) in serum	GDF15 (pg/ml) in CSF
Total cohort	390 (100)	561.01 ± 552.78**	146.84 ± 111.88**
Males	161 (41.3)	587.64 ± 621.97	163.16 ± 123.83*
Females	229 (58.7)	542.28 ± 499.02	135.38 ± 101.36*
BMI < 25 kg/m^2^	161 (41.3)	544.43 ± 555.57*	140.80 ± 123.29*
BMI ≥ 25 kg/m^2^	229 (58.7)	572.67 ± 551.74*	151.09 ± 103.17*
Females on contraceptives	19 (8.3)	396.41 ± 163.07	94.21 ± 87.87**
Females without contraceptives	210 (91.7)	555.48 ± 516.97	139.10 ± 101.86**
Smoking – yes	27 (6.9)	498.88 ± 244.30	138.08 ± 96.16
Smoking – none	363 (93.1)	565.63 ± 569.00	147.50 ± 113.05
Hypertension – yes	105 (26.9)	819.40 ± 710.22**	214.50 ± 140.66**
Hypertension – none	285 (73.1)	465.81 ± 447.27**	121.92 ± 87.08**
Type 2 diabetes – yes	30 (7.7)	1153.73 ± 804.89**	248.96 ± 167.80**
Type 2 diabetes – none	360 (92.3)	511.62 ± 496.96**	138.33 ± 101.71**
Disease groups	n (%)	GDF15 (pg/ml) in serum	GDF15 (pg/ml) in CSF
Controls	170 (43.6)	491.16 ± 359.91	128.22 ± 92.52
Multiple sclerosis	68 (17.4)	406.73 ± 166.27	94.47 ± 37.30
Epilepsy	50 (12.8)	663.31 ± 803.82	168.59 ± 110.73
Cerebrovascular disease	46 (11.8)	870.03 ± 919.70 ^**#**^	197.24 ± 124.91 ^**#**^
Pseudotumor cerebri	26 (6.7)	444.32 ± 256.37	141.35 ± 90.85
Infectious disease	30 (7.7)	763.33 ± 714.61	262.34 ± 188.74 ^**#**^
CSF groups			
CSF cell count < 5 cells	330 (84.6)	556.88 ± 552.03	140.23 ± 100.71*
CSF cell count ≥ 5 cells	60 (15.4)	583.75 ± 561.03	183.24 ± 156.14*
Oligoclonal bands: yes	66 (16.9)	514.67 ± 508.25	129.35 ± 109.53*
Oligoclonal bands: none	302 (77.4) ^&^	578.92 ± 577.76	152.99 ± 114.88*
BBB dysfunction: none	336 (86.2)	507.03 ± 449.45	130.25 ± 88.62
BBB dysfunction: mild	38 (9.7)	808.68 ± 950.14	188.60 ± 99.05**°°**
BBB dysfunction: moderate	10 (2.6)	1062.44 ± 711.70**°**	406.73 ± 227.23**°°**
BBB dysfunction: severe	6 (1.5)	1179.75 ± 1011.29	378.24 ± 225.81**°**

(n = 390) and quantification of GDF15 levels in serum and CSF (cerebrospinal fluid) by ELISA**.** BBB, blood–brain-barrier; BMI, body mass index; CSF, cerebrospinal fluid; GDF15 (growth/differentiation factor-15), SD, standard deviation. * *P* < 0.05; ** *P* < 0.001; ^**#**^ *P* < 0.05 vs. Controls; **°** *P* < 0.05 vs. no BBB dysfunction; **°°** *P* < 0.001 vs. no BBB dysfunction. ^&^ data not available in 22 participants.

### Correlations of GDF15 concentrations in serum and CSF with anthropometric and biochemical parameters

**Fig. 3:**
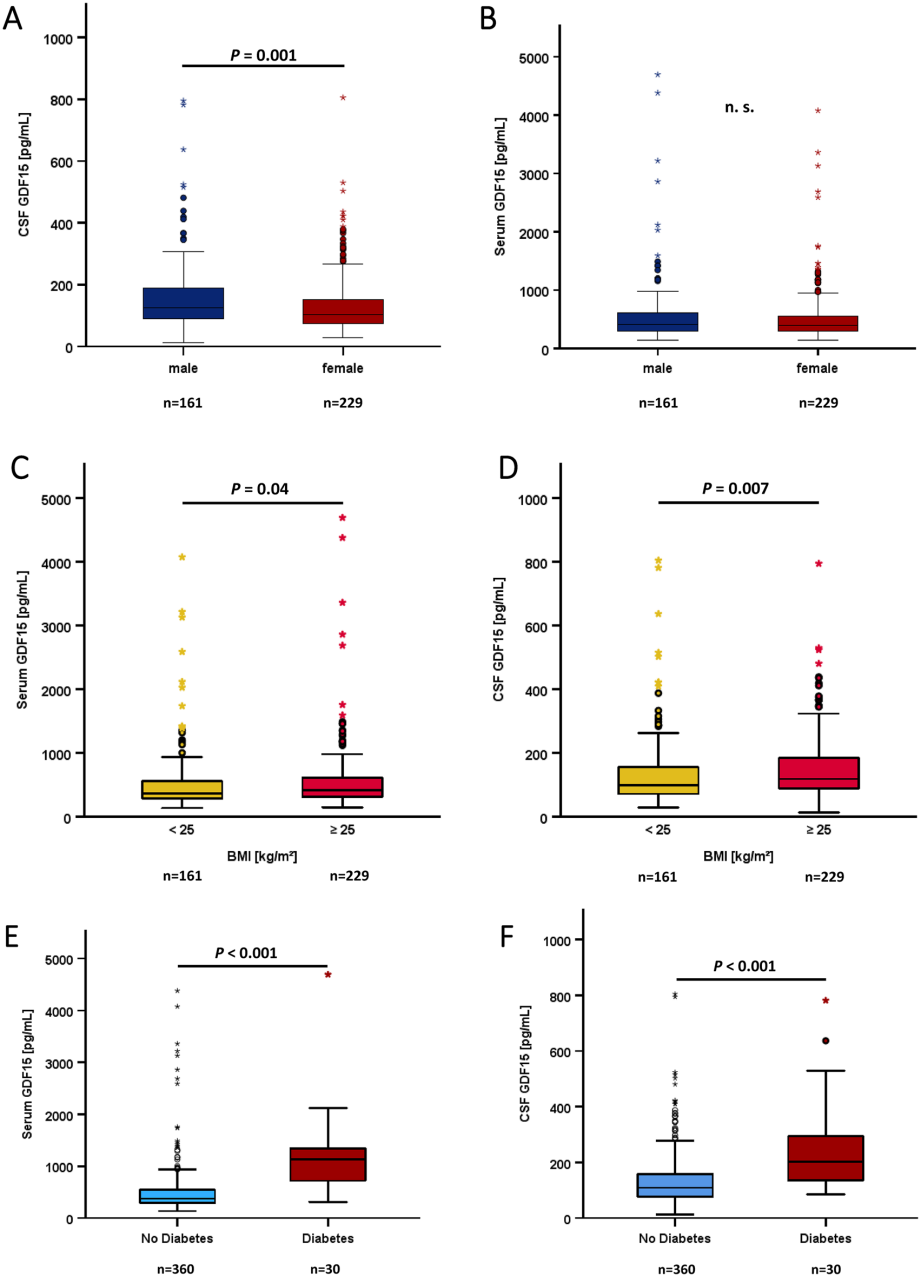
Dependence of GDF15 concentrations on gender, obesity and type 2 diabetes. **Panel A** GDF15 concentrations in CSF are higher in males than in females. **Panel B** GDF15 concentrations in serum show no gender-specific effect. **Panel C** GDF15 concentrations in serum are higher in overweight/obese patients. **Panel D** GDF15 concentrations in CSF are higher in overweight/obese patients. **Panel E** GDF15 concentrations in serum are higher in type 2 diabetic patients. **Panel F** GDF15 concentrations in CSF are higher in type 2 diabetic patients. The Mann-Whitney U test was applied.

Both, CSF as well as serum GDF15 quantities were significantly and positively correlated with age (Fig. 2 B, C). The classical CSF/serum ratio of albumin as a parameter of BBB permeability physiologically increases with age (continuous impairment of BBB during ageing). We analyzed this in the present cohort and confirmed the significant (*P* < 0.001) and positive correlation (*rho* = 0.596) between age and CSF/serum albumin ratio (data not shown). Males had higher GDF15 levels in CSF than females, whereas GDF15 in serum did not show a sexual dimorphism (Fig. 3A, B). Interestingly, females on contraceptives had significantly lower GDF15 concentrations in CSF when compared to females without contraceptives (Table 1). Since this contraceptive effect could account for the gender-specific effect in general, we additionally compared males vs. females without usage of contraceptives and found that the gender-specific effect on GDF15 levels in CSF remained significant (*P* = 0.011).

Mean GDF15 concentrations in serum and CSF were significantly higher in overweight/obese patients (BMI ≥ 25 kg/m^2^) when compared to normal body weight (BMI < 25 kg/m^2^) (Fig. 3C, D). Even more pronounced, GDF15 concentrations in serum and CSF were significantly higher in type 2 diabetic patients when compared to non-diabetic individuals (Fig. 3E, F) in the entire study cohort. This observation was also significant when analyzing solely the control group of participants (data not shown).

**Fig. 4:**
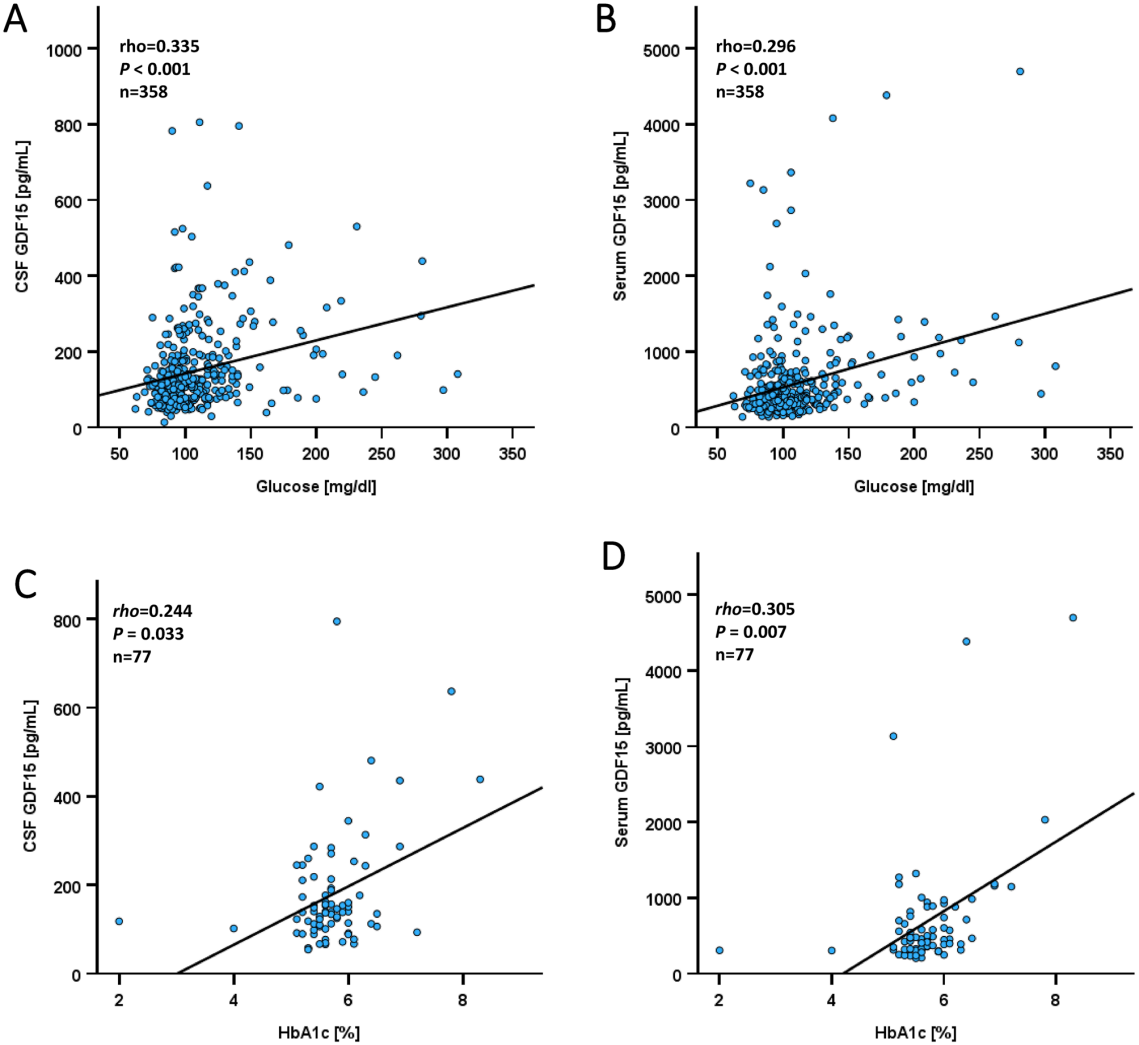
Correlation analysis (scatter plot diagrams) of CSF and serum concentrations of GDF15 with serum glucose and HbA_1c_ levels. **Panel A** Positive correlation of GDF15 concentrations in CSF with serum glucose. **Panel B** Positive correlation of GDF15 concentrations in serum with serum glucose. **Panel C** Positive correlation of GDF15 concentrations in CSF with HbA_1c_ levels. **Panel D** Positive correlation of GDF15 concentrations in serum with HbA_1c_ levels. The Spearman-rho rank correlation test was applied.

Due to this relation of GDF15 with type 2 diabetes, we aimed to calculate specific correlations of glycemic parameters such as random serum glucose concentrations and HbA_1c_ levels with GDF15. Impressively, GDF15 concentrations in CSF and serum (Fig. 4A, B) were significantly (*P* < 0.001) and positively correlated with serum glucose levels. Moreover, GDF15 concentrations in CSF and serum (Fig. 4C, D) were significantly (*P* = 0.033 and *P* = 0.007, respectively) and positively correlated with HbA_1c_ levels. Taken together, obesity, presence of type 2 diabetes, serum glucose concentrations, and HbA_1c_ levels are correlated with higher GDF15 concentrations in serum and CSF. These observations indicate an important role of obesity and carbohydrate metabolism in the regulation of GDF15 brain permeability. As a finding of questionable relevance, patients with hypertension had elevated CSF and serum levels of GDF15 (Table 1).

### Correlations of GDF15 concentrations in serum and CSF with neurological disease subgroups

**Fig. 5:**
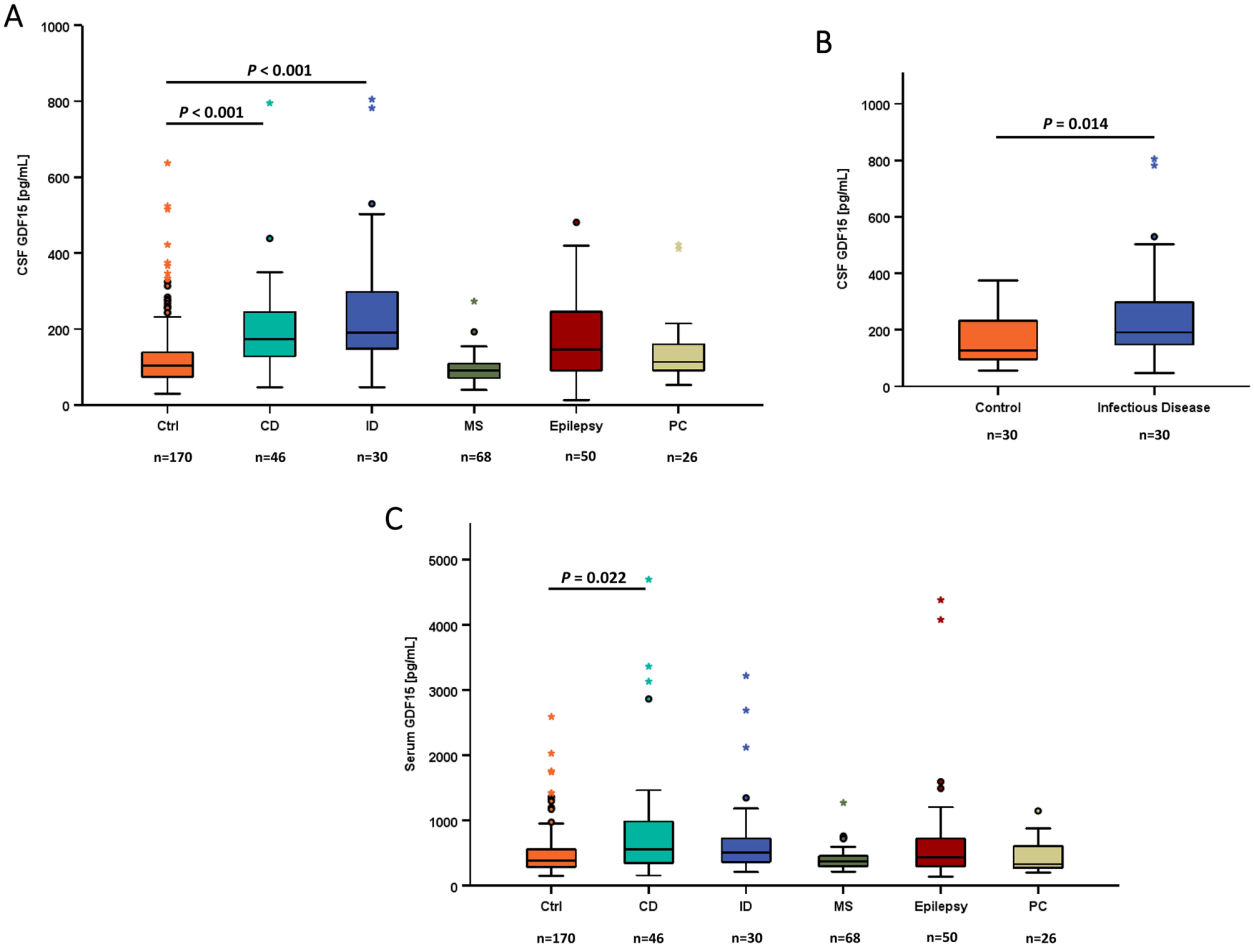
GDF15 in neurological disease groups. **Panel A** GDF15 concentrations in CSF are higher in patients with infectious diseases (and cardiovascular diseases) in the entire study cohort. The Kruskal-Wallis test was performed. **Panel B** GDF15 concentrations in CSF are higher in patients with infectious diseases when compared to matched controls. The Mann-Whitney U test was applied. **Panel C** GDF 15 concentrations in serum are not correlated to infectious diseases but only to cardiovascular diseases. Ctrl, control group; CD, vascular diseases; ID, infectious diseases; MS, multiple sclerosis; PC, pseudotumor cerebri. The Kruskal-Wallis test was performed.

We pre-specified 5 subgroups of neurological diseases and a control cohort without any evidence of neurological disease. It was the aim to investigate whether GDF15 in CSF might represent a potential biomarker for neurological diseases or a candidate for a pathophysiologic role in neurologic disorders. As depicted in Fig. 5A (for exact mean values please refer also to Table 1), CSF concentrations of GDF15 were significantly elevated in vascular diseases and infectious diseases (*P* < 0.001) when compared to the control group. In order to exclude any confounding variables, we defined n = 30 of age-, BMI-, and sex-matched controls (with a CSF/serum albumin ratio of < (4 + age/15) × 10^–3^ as commonly suggested^26^) for comparison with n = 30 patients suffering from infectious diseases. As shown in Fig. 5B, the significance of higher GDF15 concentrations in CSF of patients with infectious diseases compared to matched controls remained preserved. This was not the case for vascular diseases (data not shown). GDF15 concentrations in serum were not correlated to infectious diseases but only to vascular diseases (Fig. 5C).

**Fig. 6:**
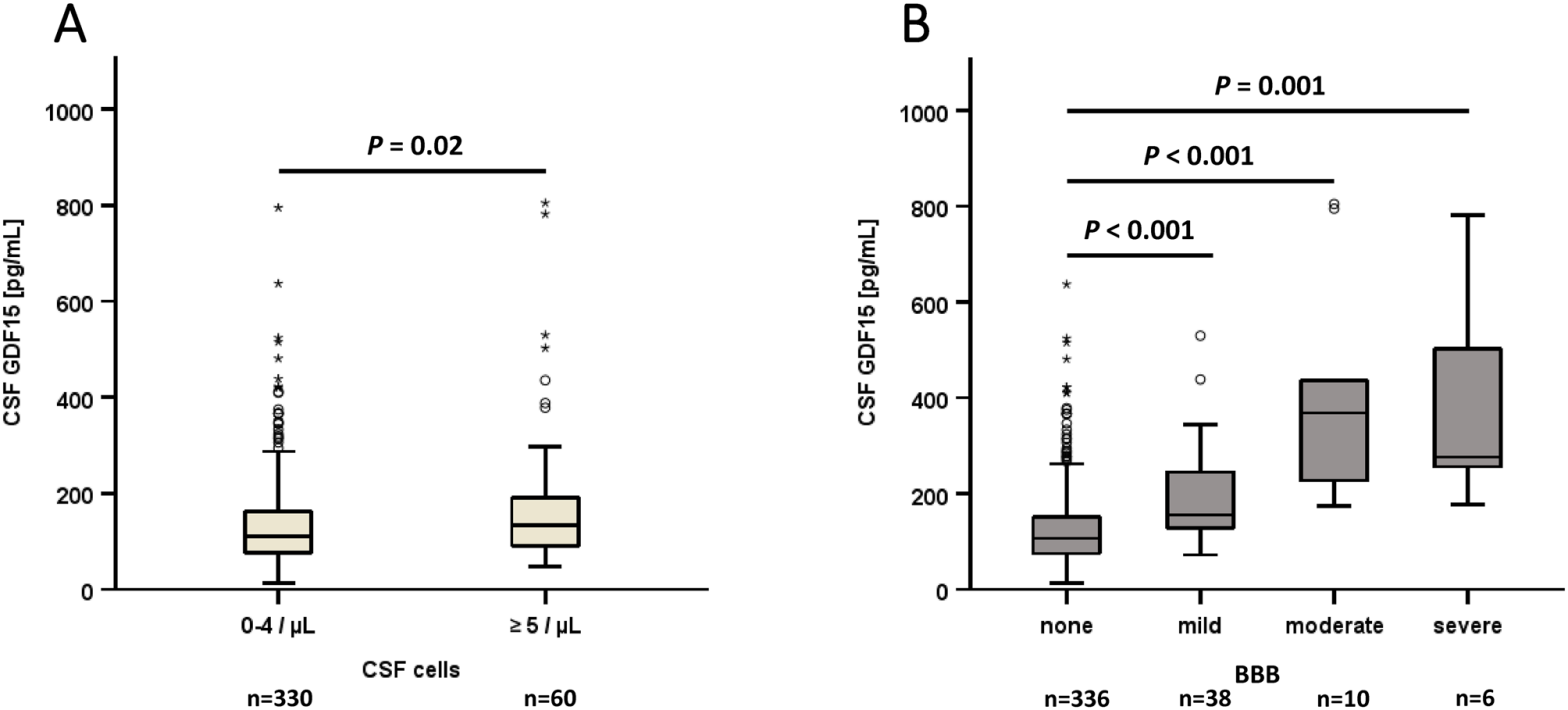
GDF15 concentrations in CSF and their relation to CSF cell count and dysfunction of the blood–brain-barrier (BBB). **Panel A** GDF15 concentrations in CSF are higher in patients with elevated CSF cell count. The Mann-Whitney U test was applied. **Panel B** GDF15 concentrations in CSF are higher in patients with dysfunction of BBB. The Kruskal-Wallis test was performed.

Taken together, GDF15 levels in CSF (but not in serum) seem to correlate specifically with infectious diseases of the central nervous system. In this context, we measured CSF GDF15 in two subgroups of patients, with a CSF cell count either < 5 cells (n = 330) or ≥ 5 cells (n = 60) (Fig. 6A). GDF15 concentrations were significantly higher in the subgroup of patients with an elevated cell count (*P* = 0.02). In this context, it seems worth to mention that GDF15 in CSF positively correlated with serum C-reactive protein levels (*P* < 0.001; *rho* = 0.234) but not with white blood cell count. Moreover, we analyzed CSF GDF15 levels in the four subgroups of patients with a differing grade of BBB dysfunction (none, mild, moderate, severe) (Fig. 6B). GDF15 concentrations were substantially elevated in mild, moderate, and severe BBB dysfunction when compared to individuals with normal BBB function (*P* < 0.001 and *P* = 0.001, respectively). Patients with presence of oligoclonal bands in CSF were characterized by lower CSF GDF15 concentrations (Table 1).

## Discussion

**Fig. 7:**
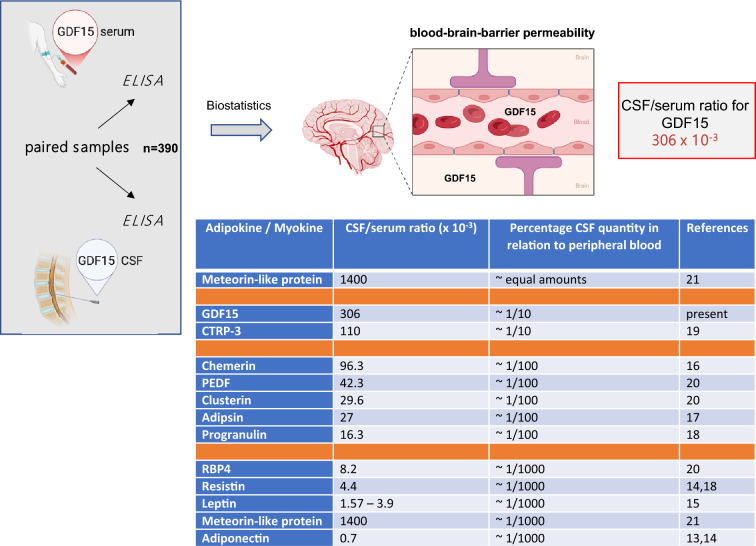
Clinical procedure and calculation of specific CSF/serum values for GDF15. BBB permeability of GDF15 and its relation to other classical adipokines is given at a glance. The higher the CSF/serum ratio, the higher the grade of permeability to the brain. Due to the percentage of the CSF protein quantity in relation to the quantity in peripheral blood serum, three grades of permeability (1/1000; 1/100; 1/10) can be defined for a better clinical undertanding. The famous, adipocyte-specific, satiety hormone leptin signals to the brain and has a quantitiy in CSF of 1/1000 in relation to peripheral blood serum. BBB, blood–brain-barrier; CSF, cerebrospinal fluid. Created with a *BioRender* personal account.

GDF15 has been discussed as potential biomarker or even pathophysiological key player in various neurological diseases, mitochondrial diseases, neuro-inflammation, metabolic stress activation, pregnancy-induced nausea, and obesity/weight regulation. In addition to peripheral tissues, the most important site of CNS-located GDF15 synthesis is the choroid plexus which secretes the protein into the CSF from where it penetrates the brain tissue through the ependymal layer^27,28^. The autochthonous production of GDF15 in the choroid plexus and the subsequent secretion into the CSF could contribute significantly to the concentrations measured in the CSF. Therefore, GDF15 in CSF might result from a combination of autochthonous expression and BBB permeability for serum GDF15. As provided by the *Human Protein Atlas* (https://www.proteinatlas.org/ENSG00000130513-GDF15/tissue), GDF15 mRNA expression is detectable in multiple organs. Whereas expression levels are highest in kidney, urinary bladder and choroid plexus, total adipose tissue expression ranges on the 10^th^ place. The present data is of clinical importance with respect to the interpretation of the aforementioned expression data regarding the choroid plexus. If the autochthonous expression of GDF15 within the choroid plexus would represent the main source of GDF15 concentrations in CSF, one would not expect a significant and positive correlation between serum and CSF levels. However, data on GDF15 concentrations in human CSF is sparse and detailed studies on CSF/serum ratios of GDF15 in deeply characterized and larger patient populations are completely lacking. We could demonstrate a strong (*P* < 0.001) and positive correlation between serum and CSF GDF15 concentrations and a relatively high CSF/serum ratio of 306 × 10^–3^ indicating that BBB permeability for GDF15 is a main and additional component determining CSF concentrations. In accordance with this, GDF15 CSF/serum ratio is positively correlated with albumin CSF/serum ratio. These present observations strongly argue against a sole or predominant role of the choroid plexus in determining GDF15 CSF concentrations. The grade of BBB permeability for GDF15 is very high when compared to other adipokines. Among 12 classical adipokines that have been discussed as important mediators of the fat-brain-axis, we found that GDF15 has the second highest grade of permeability to the brain (after meteorin-like protein). Figure 7 finally illustrates the experimental approach of the present study and summarizes CSF/serum ratios for classical adipokines/myokines. For example, the highly adipocyte-specific secretory hormones leptin (named “satiety hormone”) and adiponectin are characterized by relatively low CSF/serum ratios (CSF concentrations about 1/1000 of the corresponding serum concentrations). In contrast to these classical adipokines, the CSF/serum ratio of GDF15 is relatively high.

In humans, the hyperglycemia-related compound of the metabolic syndrome complex is related to elevated serum GDF15 levels^29^. A murine GDF15 knockout model reported increased adiposity and food intake together with reduced energy expenditure and physical activity^30^. We could describe an impressive, positive correlation of CSF and serum GDF15 concentrations with parameters of carbohydrate metabolism such as serum glucose and HbA_1c_. Moreover, overweight and type 2 diabetic patients had significantly higher levels of GDF15 in serum and CSF. These observations indicate that central nervous actions of GDF15 (that depend on the CSF concentrations) seem to be co-regulated by metabolic factors such as obesity and carbohydrate metabolism. Type 2 diabetic patients and obese patients are characterized by hyperinsulinemia. In vitro, we were able to demonstrate that insulin upregulates GDF15 protein secretion in adipocytes. Future and mechanistic studies are needed to clarify the potential mechanisms of an interplay between metabolic factors and central nervous actions of GDF15.

Data on a role of GDF15 in neurological diseases are diverse, partially contradictive and difficult to interpret. GDF15 levels were higher in pediatric patients with acute infection-triggered encephalopathy when compared to complex febrile seizures^31^. In a cohort of patients with multiple sclerosis, serum GDF15 levels were lower when compared to non-inflammatory CNS diseases^32^, however, CSF levels of GDF15 were not reported in the cited study. A clinical trial reported elevated GDF15 levels in CSF of patients suffering from multiple sclerosis, especially in those with primary progressive multiple sclerosis^33^. Another study could demonstrate increased quantities of GDF15 in CSF only in patients with stable multiple sclerosis but not in multiple sclerosis in general^34^. In patients with vanishing white matter disease, GDF15 concentrations in CSF (but not in serum) were strongly elevated when compared to controls^35^. Moreover, studies found increased CSF levels of GDF15 in patients suffering from cerebral palsy^36^, Parkinson`s disease^37^, or glioblastoma^38^ Although not expressed in *post mortem* brain tissue, a large-scale proteomic approach of 4877 plasma proteins in 10.981 patients revealed a strong association of GDF15 in plasma and serum with 25-year dementia risk^39^. We aimed to investigate a large and deeply characterized cohort of patients undergoing neurological evaluation including spinal puncture in order to test GDF15 concentrations for a potential role as a biomarker in several neurological diseases. Only in the subgroup of infectious neurological diseases we could observe significantly elevated levels of GDF15 when compared to controls and even when compared to age-, BMI-, and sex-matched controls without deterioration of the BBB. Moreover, GDF15 was increased in subgroups of patients with elevated CSF cell count and/or disturbed BBB. While this study is not able to give any mechanistic and causative explanations for this observation, it seems worth to further investigate the potential role of GDF15 as a biomarker and as a possible player in infectious neurological diseases. The documented gender-specific effect is not completely convincing and should not be overinterpreted. Limitations of the present study include the mainly descriptive nature of the results and the monocentric study design. One of the main advantages of the present study is the presentation of reliable GDF15 serum and CSF concentrations in a large cohort of deeply characterized controls and patients with neurological diseases. This could be used for the definition of future normal ranges and reference values. Most importantly, we could present for the first time a reliable CSF/serum ratio for GDF15 describing its BBB permeability. This value of 306 × 10^–3^ is helpful for future studies and will facilitate classification of the permeability of GDF15 in relation to other adipokines.

## Summary

GDF15 is expressed in and secreted by differentiated adipocytes. It circulates in peripheral blood serum and crosses the blood–brain-barrier. It is highly permeable to the brain with a CSF/serum ratio of 306 × 10^–3^. GDF15 is significantly higher in overweight and type 2 diabetic patients and correlates positively with serum glucose and HbA_1c_. Insulin upregulates GDF15 secretion in adipocytes in vitro. GDF15 in CSF is elevated in patients with increased CSF cell count and impaired blood–brain-barrier function. Among five subsets of neurological diagnoses, GDF15 is specifically increased in CSF and serum of patients with infectious diseases.

## Conclusions

GDF15 represents a promising adipokine and mediator of the fat-brain-axis that is co-regulated with metabolic factors and elevated in neurological patients suffering from infectious diseases.

## Data Availability

Data are available from the corresponding author upon reasonal request.
